# Consumer Narratives in ADR Reporting: An Important Aspect of Public Health? Experiences from Reports to a Swedish Consumer Organization

**DOI:** 10.3389/fpubh.2015.00211

**Published:** 2015-09-01

**Authors:** Andreas Vilhelmsson

**Affiliations:** ^1^Division of Social Medicine and Global Health, Department of Clinical Sciences, Faculty of Medicine, Lund University, Malmö, Sweden; ^2^School of Social Work, Faculty of Social Sciences, Lund University, Malmö, Sweden

**Keywords:** public health, public policy, adverse drug reaction reporting systems, adverse drug reactions, antidepressants, post-market safety, discontinuation, adverse events

## Introduction

Harms of drugs are an important cause of mortality and morbidity. The use of pharmaceuticals always entails a risk of adverse events, often to an unknown extent, and increased drug utilization has made drug-related problems a common occurrence (1). It is, for instance, estimated that adverse drug reactions (ADRs) cause the European Union almost 200,000 deaths annually (2) and cost approximately €79 billion (3).

The World Health Organization (WHO) clearly recognizes the need for prescribed medicines to be of good quality, safe, effective, and used by the right patient in the right dose at the right time in order to minimize the risk of harm (4). The thalidomide disaster around 1960 underscored the necessity of systems to monitor medicines safety after marketing. As a consequence, governments developed systems in order to affirm that drug products are safe and efficacious enough to be permitted on the market and that the pharmaceutical industry needed to be regulated since these companies have a responsibility to protect public health (5). This concern with patient safety and the rational usage of drugs is often referred to as pharmacovigilance (PV) (6), which can be defined as “the science and activities relating to the detection, assessment, understanding, and prevention of adverse events or any other possible drug-related problems” (7). It is however well established that knowledge about medicines safety is limited at the time of licensing of a new product and spontaneous reporting of ADRs to regulatory authorities and drug manufactures is perhaps the most important way of monitoring the post-market safety of medicines (8). National databases to collect signals of ADRs from health care professionals (HCPs) were established already from the late 1960s and a WHO international collaboration started in 1968. With their *Drug Monitoring Program*, WHO supports the reporting of potential ADRs by HCPs (9), but it is a well-recognized problem by the Organization, that this group under-report serious and fatal reactions (4). Unexpected ADRs are, for instance, seldom reported.

## Consumer Reporting

To allow the general public to report directly to the authorities, so-called consumer reporting, has been presented as an alternative way to increase ADR reporting. This is sometimes also referred to as direct patient reporting (DPR). An advantage in using the term “consumer reporting” is that it clarifies that it is referring to direct reporting from the person affected (instead of reporting within or via a health care setting) and that it is a matter of consumer rights. The term is also used by the WHO (10).

Some concerns with this kind of direct reporting have, however, been raised. First, there is the potential lack of medical confirmation that may hamper the analysis of cause and effect between the drug and the adverse event (11). Second, there have been some concerns that media potentially might influence consumer reports and thereby provide more selected reporting than from health professionals and as a consequence complicate analysis. Third, critics (mostly health professionals and regulatory agencies) previously stated that consumer reports would create “noise” and prove a drain on surveillance systems (7, 12). Despite these concerns, several research studies in the last decade or so have suggested that consumer reports instead can add value to HCP reports by identifying potential new adverse reactions (13–17) and considerably contribute to reliable PV systems (18). On the contrary to critical beliefs, subjective toxicities are instead at high risk of being under-reported by physicians, and the WHO states that consumer reporting can be an important aspect to both help the individual patient to receive optimal therapy and also to safeguard public health programs (7).

Today, around 46 countries have some kind of national consumer reporting system (18). Citizens in the US, Canada, Australia, and New Zealand have had the possibility to report ADRs directly since the 1960s, while in other countries, including Denmark, the Netherlands, the UK, and Sweden, reporting has only been available since 2003 and later. Since 2012, all EU countries are obliged to establish patient/consumer reporting within their spontaneous reporting systems as a part of the new European pharmacovigilance legislation (Directive 2010/84/EU) (Regulation 1235/2010) (19). The purpose of this legislation is to improve patient safety and public health and to further accentuate patient influence. This approach clearly indicates a change in attitude in which patients’ experiences are valued and believed to accelerate the acquisition of knowledge about ADRs. Still, the awareness that patients and consumers can report ADRs is thought to be low in most countries and there is a low awareness of available reporting systems (20). It has also been argued that many consumer reporting systems focus only on adverse events, missing out on other aspects of medicine use, such as experiences in ineffectiveness (13).

## Consumer Reporting in Sweden

In Sweden, it has only been possible for the general public to submit reports to the Medical Products Agency (MPA, the national medicines regulatory authority) since 2008. Consumers have the option to report electronically direct on the MPA website or by printing out the report scheme and send it via regular mail. The agency now regards these consumer reports to be a valuable contribution in the monitoring of safety aspects in medicines (16) despite previous concerns like the above mentioned.

Before the MPA implemented consumer reporting in Sweden, the public had since 1997 had the possibility to report ADRs to the consumer organization KILEN (Consumer Institute for Medicines and Health), working on consumer rights issues of dependence, side effects, and injuries related to medicines. This organization established a consumer database in 1997 to collect consumer reports, mainly focusing on benzodiazepines and antidepressants. Reports came mainly through personal contact by telephone and visits. This gave the possibility for consumers to report suspected ADRs and to share their experience of medicine use. In 2002, KILEN also introduced the possibility for the public to report suspected ADRs via an online report form on their webpage allowing for the reporting consumer to add free text comments of their ADR experiences. Reported drugs to KILEN were coded according to the Anatomical Therapeutic Chemical (ATC) classification and reported ADRs according to MedDRA^®^ terminology.

KILEN as a consumer institute was unexpectedly forced to cease operations in March 2007, when the Swedish Parliament (Riksdag) decided not to allow further government grants. It was however still possible to report ADRs through the web-based form to KILEN, but after a couple years as a member organization KILEN had to shut down their website in 2013.

### An analysis of KILEN consumer reports

Unfortunately, the majority of the consumer reports made to KILEN have not yet been scientifically scrutinized. The exception is the ones made to the website and an analysis of all submitted reports between the years 2002 and 2009 have shown and confirmed the importance of consumer reports as an important complement to HCP reports (21). These ADR reports have been analyzed both quantitatively (22) and qualitatively, both regarding the ADR experiences *per se* (23), but also regarding experiences of the medical encounter (24). A majority of these reports (70.5%) concerned antidepressants [mostly selective serotonin-reuptake inhibitors (SSRIs)]. Other research studies on spontaneous reporting systems have shown that ADRs from antidepressants are commonly reported (13, 25).

Apart from giving a vivid description of users’ symptoms and suspected adverse reactions, the KILEN reports also indicated how these experiences affected their daily lives (21). Many of the consumer reports contained descriptions of experiences of severe psychiatric adverse effects with antidepressant treatment. This was especially apparent during discontinuation. This is not exclusive for the KILEN reports, but has been found in other reporting systems as well, for instance the UK Yellow Card Scheme where patients were more likely than HCPs to include information about symptoms and the impact it had on them (15). The UK system also identified new “serious” reactions not already included in the summary of product characteristics (SPCs), which was quite apparent in the KILEN consumer reports as well (22). Experiencing a “sensation of unreality” was, for instance, a common psychiatric ADR reported to KILEN and not listed at all as an ADR in the SPC and numerous KILEN narratives indicated patients experiencing feeling like a “zombie” incapable of having or sharing feelings toward others, including family members.

### The problem of discontinuation

The KILEN consumer reports contained other important findings as well. Almost one-third of all the psychiatric ADRs reported happened during discontinuation treatment (mean 30.6%). These symptoms were often described as being particularly severe and problematic. This result is quite congruent with other research, where abrupt cessation of SSRIs is argued to produce withdrawal symptoms in up to one-third of the patients (26). Interestingly, discontinuation symptoms from the KILEN reports were not always mentioned in the Swedish SPC or regarded as rare. This is an important finding since discontinuing symptoms can be regarded by the treating physician as a relapse of the original disorder (27), which in turn can lead to continued drug treatment or an increase in dosage. Hence, we encounter a problem of interpretation; the reported ADR may one the hand occur as a symptom of the illness for which the antidepressant had been prescribed in the first place, or it may be a signal of a potential severe ADR. These kinds of problems were quite apparent in the KILEN narratives that included reports of consumers perceiving being dismissed by their doctor regarding the authenticity of their experienced discontinuation symptoms. Similar dismissive attitudes among HCPs have been reported in the UK’s Yellow Card Scheme as well (28).

### Anecdotal and non-scientific reports?

As previously mentioned, there is the question of validity of consumer reports. In the past, these reports were mostly dismissed as anecdotal or non-scientific (29), but research and experience have shown that patients and consumers can in fact distinguish between suspected ADRs and other symptoms (30). Consumers have also been shown to be not only capable of describing their experiences but also balance the benefits and burden of treatment (13). What the KILEN narratives adds is that they clearly indicate that consumer experiences may provide important insight and ought not to be so easily rejected, but that it also reveals how patients communicate with their doctors, and how patients often feel that doctors will not listen. Physicians with good communication skills will probably not only be able to detect potential ADR problems earlier but also stimulate ADR reporting and gathering of information. By preventing medical crises and expensive interventions, the treating physician can thereby provide better support to their patient (31).

## Consumer Reporting and Public Health

Consumer reports are not only important for the individual but also can have great significance for public health as well. By collecting as many experiences as possible, new suspected ADRs can be detected, meaning that it would be easier to analyze potential causation on population basis and thereby preventing unnecessary suffering. Public health is built on trust and harms to even just a few patients can destroy the credibility and success of an important public health program (32). As previously argued by others, combining all reports regardless of reporter type is recommended since it yields the largest critical mass of reports for signal detection (16). Further, recognition of these consumer reported ADRs could prevent misdiagnosis and the worsening of potentially severe iatrogenic disorders.

When reports contain insufficient details, then doctors and patients have a false sense of security about the safety of the product that they are prescribing and using. Here, consumer reports can be of special importance since they describe the burden of ADRs for individuals, which is a major health component that is missing from public health estimates of disease burden in populations (33).

## The Importance for Future Analysis and Promotion

The Swedish MPA consumer reporting system developed slowly with ~400 reports from the public in 2008 to 1,360 in 2014, with the expectation of a major uneven distribution due to the vaccination campaign during the A(H1N1) pandemic in 2009, resulting in 2,541 reports that year. In total, the agency has received over 7,500 reports from the general public, containing not only a description of the symptom of a suspected ADR but also the user’s description in free text. However, these regarding ports have not been adequately compiled over time or qualitatively analyzed, missing out on the consumer experience given in free text. This is most unfortunate since the KILEN reports has shown that consumer reports may contribute with significant information since these reports also include how the medicine is affecting the user in his or her daily life (21). The MPA reports ought therefore to be compiled and analyzed in the same way as, for instance, the Danish and UK national PV systems have been scrutinized (14, 15).

Unfortunately, the MPA has not promoted and informed the general public about the possibility to report suspected ADRs directly to the national agency much. Hence, it is imperative that consumer reporting is actively promoted to the general public as soon as possible. Not only via, for instance, the official websites of drug regulatory agencies and drug product information leaflet (PIL) but also through public information. As indicated by the WHO, consumer reporting should be as easy and cheap as possible with, for instance, easy access to prepaid reporting forms (10). We know that consumer reporting increases the number of ADRs reported to PV centers and contributes to signal detection (16), and consumers aware of ADR self-reporting systems appear prepared to use them, but promotion of and education on how to use them are required (20). This could mean for instance active promotion and raising awareness at local pharmacies through brochures and information when a medicine [both prescription and over-the-counter (OTC)] is purchased. These brochures could also include easy accessible information of already collected and analyzed consumer reports. In our digital age, the use of available social media data for ADR monitoring is being increasingly discussed by researchers (34). The use of social media and especially Facebook has been suggested as one way to increase spontaneous ADR reporting (35). There are even specific apps, for instance MedWatcher, available for reporting side effects of drugs, vaccines, and medical devices (36). These are all positive developments since there is also a need for more consumers to report their experienced suspected ADRs.

In conclusion, if drug regulatory agencies truly want the general public to report and share their ADR experiences through their reporting systems, they must (1) inform and promote and inform the public about their possibility to report suspected ADRs, and (2) adequately analyze all consumer reports, including the free text, in order to monitor the drugs used and to detect and confirm new ADRs as soon as possible. The public need some kind of feedback that their experiences are being taken seriously and that their effort is not being taken for granted. Otherwise, authorities cannot expect or rely on their citizens to contribute to these kinds of systems. Thereby, we risk missing out on an important aspect of public health, that is, monitoring the post-market safety of medicines.

## Conflict of Interest Statement

The author declares that the research was conducted in the absence of any commercial or financial relationships that could be construed as a potential conflict of interest.

## Funding

No sources of funding were used to assist in the preparation of this manuscript.
